# The Nonribosomal Peptide Valinomycin: From Discovery to Bioactivity and Biosynthesis

**DOI:** 10.3390/microorganisms9040780

**Published:** 2021-04-08

**Authors:** Shuhui Huang, Yushi Liu, Wan-Qiu Liu, Peter Neubauer, Jian Li

**Affiliations:** 1 School of Physical Science and Technology, ShanghaiTech University, Shanghai 201210, China; huangshh@shanghaitech.edu.cn (S.H.); liuysh1@shanghaitech.edu.cn (Y.L.); liuwq@shanghaitech.edu.cn (W.-Q.L.); 2 Chair of Bioprocess Engineering, Department of Biotechnology, Technische Universität Berlin, 13355 Berlin, Germany

**Keywords:** valinomycin, nonribiosomal peptide, bioactivities, biosynthesis, *Streptomyces*, heterologous production, cell-free biosynthesis

## Abstract

Valinomycin is a nonribosomal peptide that was discovered from *Streptomyces* in 1955. Over the past more than six decades, it has received continuous attention due to its special chemical structure and broad biological activities. Although many research papers have been published on valinomycin, there has not yet been a comprehensive review that summarizes the diverse studies ranging from structural characterization, biogenesis, and bioactivity to the identification of biosynthetic gene clusters and heterologous biosynthesis. In this review, we aim to provide an overview of valinomycin to address this gap, covering from 1955 to 2020. First, we introduce the chemical structure of valinomycin together with its chemical properties. Then, we summarize the broad spectrum of bioactivities of valinomycin. Finally, we describe the valinomycin biosynthetic gene cluster and reconstituted biosynthesis of valinomycin. With that, we discuss possible opportunities for the future research and development of valinomycin.

## 1. Introduction

Nature, like a magic chemist, is able to synthesize complex compounds from simple building blocks. These compounds are called natural products (NPs), whose diverse chemical structures usually confer significant biological and pharmaceutical activities to them [1]. Therefore, NPs have historically served as an abundant source for potent drugs to combat human diseases such as pain, infections, and cancers [2]. During the past two centuries, tens of thousands of NPs have been isolated and characterized from various living organisms (e.g., microorganisms, plants, and animals) [3]. Among the large number of NPs, nonribosomal peptides (NRPs) belong to an important class of peptide compounds, which are synthesized through mRNA-independent assembly lines termed nonribosomal peptide synthetases (NRPSs) [4,5]. NRPSs are giant multimodular enzymes with molecular weights often ranging from one hundred to several hundreds of kilodaltons (kDa). Usually, one typical NRPS module contains three core domains, which are an adenylation (A) domain (~50 kDa) for substrate (monomer/building block) selection and activation, a thiolation (T) domain (8–10 kDa) for substrate and growing peptidyl chain tethering, and a condensation (C) domain (~50 kDa) for peptide bond formation. Once all building blocks are incorporated into the final full-length peptide chain, a thioesterase (TE) domain (~35 kDa) located furthest downstream in the last module catalyzes the release of the final product. In addition, some optional domains, for example, epimerase (E), ketoreductase (KR), and methyltransferase (MT), also present in NRPS modules to perform modifications of monomeric building blocks. To date, more than 500 variant monomers for NRPS assembly lines have been identified including proteinogenic and nonproteinogenic amino acids and other carboxylic acids (e.g., aryl acids) [6,7]. As a consequence, the NRP structures are remarkably diverse and complex, which leads to a high density of functional groups and contributes notably to the observed pharmaceutical properties [8,9]. The most well-known examples of NRP antibiotics are the penicillin and cephalosporin families [10], as well as the vancomycin [11], which are still being used daily in clinic.

**Figure 1:**
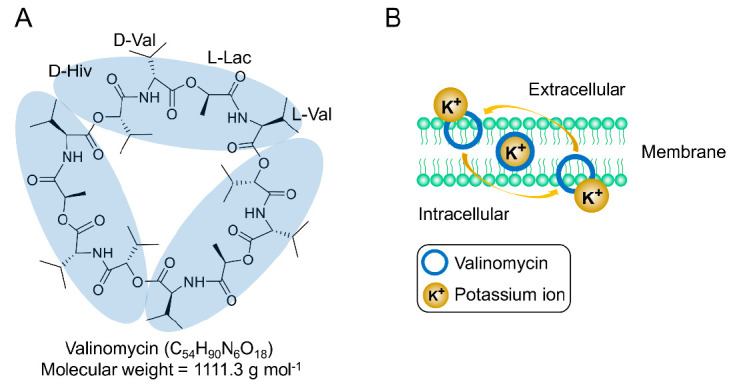
(**A**) Chemical structure of valinomycin and (**B**) valinomycin acts as a potassium-specific ionophore.

Valinomycin is an NRP compound that was first isolated and characterized 65 years ago from *Streptomyces fulvissimus* [12]. Two years later, in 1957, Brockmann and Geeren totally hydrolyzed valinomycin and proposed the structure of valinomycin as a 24-membered cyclic peptide cyclo-(d-α-hydroxyisovaleryl-d-valyl-l-lactyl-l-valyl)_2_ [13]. However, subsequent work indicated that the correct chemical structure of valinomycin was a 36-membered cyclododecadepsipeptide, which consists of a triple repeating unit of d-α-hydroxyisovaleryl-d-valyl-l-lactyl-l-valyl with a molecular weight of 1111.3 g mol^−1^ (C_54_H_90_N_6_O_18_) [14,15]. Clearly, the building blocks of valinomycin include not only two proteinogenic and nonproteinogenic amino acids (l-valine and d-valine), but also two carboxylic acids (d-α-hydroxyisovaleric acid and l-lactic acid). As shown in the structural formula (Figure 1A), valinomycin as a cyclodepsipeptide is composed of alternating peptide and ester linkages between each residue [16]. This structure conformation forms a hydrophobic surface and a polar cavity in which one potassium ion (K^+^) can be coordinated with the six oxygen atoms of the interior ester carbonyls, forming a valinomycin-K^+^ complex [17,18,19,20]. The size of the cavity is suitable for accommodating a K^+^ ion but not for other metal ions (e.g., Na^+^ and Li^+^), which makes valinomycin a potassium-specific ionophore and the resulting bioactivities are connected to it. Importantly, crystal structures of valinomycin have been known for a long time [21,22]. Its polymorphism would have to be taken into account in a possible manufacturing of drug formulations, as discussed in a report [23]. Previous studies on the mechanism of action of valinomycin have demonstrated that, due to the hydrophobic surface of valinomycin, the valinomycin-K^+^ complex can be incorporated into biological bilayer membranes and allows the transportation of K^+^ through the membrane to destroy the normal K^+^ gradient across the membrane, thus dissipating the membrane potential, and as a result kills the cells [24,25,26,27,28,29]. The working mechanism of valinomycin as a potassium ionophore in the biological membrane is illustrated in Figure 1B. Structure–activity relationships studied with valinomycin analogs showed that the cyclic 12-residue peptide is critical for the bioactivities of valinomycin. Alteration of the valinomycin structure by changing the ring size or amino acid residues significantly reduces the capacity to form a stable valinomycin-K^+^ complex and, consequently, its antimicrobial activity [30,31,32]. While numerous studies have revealed that the effect of valinomycin on cells is primarily due to the dissipation of membrane potential, inhibition of protein synthesis at the level of elongation was proven to be another mode of action of valinomycin [33,34,35,36]. Moreover, recent evidence also indicated that the cellular response to valinomycin is very complex and involves an intricate network of proteins related to mitochondria, vacuoles, and other membrane compartments [37].

**Figure 2:**
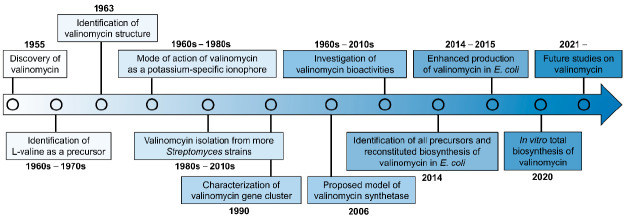
The timeline of valinomycin researches.

Since its discovery in 1955, continuous studies on valinomycin have been carried out from early structure characterization, chemical synthesis, biogenesis, and bioactivity (mechanism of action) to recent biosynthetic gene cluster identification and heterologous in vivo/in vitro production (see a research timeline of valinomycin in Figure 2). Although many research papers of valinomycin have been published so far, a comprehensive review with a focus on valinomycin is still lacking. Therefore, this motivates us to comprehensively summarize valinomycin, ranging from its discovery and characterization to bioactivity and biosynthesis. To this end, we first briefly introduce the chemical structure and related features of valinomyicn, as demonstrated above. In the following sections, we then summarize many documented bioactivities of valinomycin. Finally, we describe in detail the progress made in characterizing the valinomycin biosynthetic gene cluster (i.e., the NRPS valinomycin synthetase), and the production of valinomycin in native producers as well as reconstituted biosynthesis of valinomycin in vivo and in vitro.

## 2. Biological Activities of Valinomycin

Valinomycin was initially isolated as an antibiotic compound, showing the antibacterial activity against *Mycobacterium tuberculosis* [12]. It was also the first natural compound recognized as an ionophore with antibiotic activity [38]. Later, a diverse spectrum of biological activities of valinomycin was demonstrated that ranges from antifungal, antiviral, and insecticidal to antitumor efficacy. Recent studies even reported that valinomycin as a mitophagy activator also plays a positive role in the treatment of Parkinson’s disease [39] and Alzheimer’s disease [40]. Examples of such bioactivities are introduced in this section and representative dose-dependent activities are summarized in Table 1.

### 2.1. Antibacterial Activity

Valinomycin shows a wide range of antibacterial activity. The inhibition of cell growth was extensively tested on a series of bacteria, including Gram-positive bacteria, for example, *Staphylococcus aureus*, *Staphylococcus faecalis*, *Streptococcus pyogenes*, *Clostridium sporogenes*, *Listeria innocua*, *Bacillus subtilis*, *Enterococcus faecalis*, and *Micrococcus luteus*, as well as Gram-negative bacteria like *Escherichia coli*, *Salmonella enterica*, *Enterobacter cloacae*, *Neisseria gonorrhoeae*, and *Stenotrophomonas maltophilia* [29,41,42,43,44]. These studies indicated that, although to different levels, valinomycin showed growth inhibition against all tested Gram-positive bacteria. However, none of the selected Gram-negative bacteria were inhibited by valinomycin. The lack of susceptibility of Gram-negative bacteria to valinomycin is largely attributed to their outer membrane of the cell wall, serving as a selective barrier and limiting the intracellular access of an antibiotic [43,45]. This was also demonstrated by a previous report that if Gram-negative *E. coli* cells were treated with Tris/EDTA, the cells then became more permeable and sensitive to valinomycin [46]. The antibacterial activity of valinomycin was found to be dependent on the pH value and K^+^ concentration in the cultivation medium. While valinomycin displayed activity at a relatively broad pH range, significant inhibition of bacterial growth was observed mainly at alkaline pH values (e.g., pH 8.5) [29]. Previous studies also demonstrated that, in a medium with a low K^+^ concentration, valinomycin remarkably inhibited bacterial cell growth [41,44]. For example, when *Streptococcus pyogenes* was cultivated in Tryptose Broth (TB) with a low K^+^ concentration (3 mM), the minimum inhibitory concentration (MIC) for *S. pyogenes* was 0.02 μg/mL. However, by switching the cultivation to Trypticase Soy Broth (TSB) with a high concentration of K^+^ (35 mM), the MIC was notably increased to 1 μg/mL. This is interesting, as the inhibitory effect of valinomycin can be largely reversed by addition of excess K^+^ to the medium. The antibacterial action of valinomycin was ascribed to the ionophore-mediated loss of K^+^ from the bacterial cell, leading to the impairment of protein synthesis [41,44].

### 2.2. Antifungal Activity

Valinomycin also possesses antifungal activity against different fungi including human and plant pathogens. Pettit et al. [42] reported that valinomycin inhibited the growth of the two human pathogenic fungi *Candida albicans* and *Cryptococcus neoformans* in disk diffusion assays with MICs of 0.39–0.78 μg/disk and 50–100 μg/disk, respectively. The fungistatic spectrum of valinomycin on various plant pathogenic fungi was also examined. For instance, valinomycin exhibited antifungal activity against *Phytophthora capsici*, a highly destructive pathogen of vegetables, with a half-maximal inhibitory concentration (IC_50_) of 15.9 μg/mL [47]. In another study, the growth of two plant pathogens, *Botrytis cinerea* (gray mold) and *Magnaporthe grisea* (rice blast), were strongly inhibited by valinomycin [48]. Germination of their spores was completely inhibited at a MIC of 4 μg/mL. The mycelial growth of *B. cinerea* and *M. grisea* was also inhibited by valinomycin with half-maximal effective concentrations (EC_50_) of 5.2 and 4.3 μg/mL, respectively. In addition, in vivo control efficacy tests indicated that valinomycin can be an effective control agent for the *Botrytis* disease in cucumber plants, with a comparable efficiency as the commercial fungicide vinclozolin [48]. This suggests that valinomycin might be a potential antifungal agent for the control of *Botrytis* diseases in agriculture. Furthermore, valinomycin displays antifungal activity against other fungal species such as *Aspergillus niger*, *Fusarium graminearum*, *Sclerotinia minor*, *Penicillium verrucosum*, and *Rhizoctonia solani* [49,50,51]. However, the antifungal mechanism of valinomycin was not elucidated in these above-mentioned studies. Using two representative fungi *Candida albicans* and *Cryptococcus albidus*, Makarasen and colleagues investigated the mode of action and verified the efficacy of valinomycin alone and in combination with an antifungal antibiotic amphotericin B (AmB) against both fungi. They found that valinomycin in combination with AmB can promote the permeability of the fungal cell wall and the cell membrane and may penetrate into the cell wall and inhibit ergosterol formation, eventually leading to cell death [52].

### 2.3. Antiviral Activity

In human history, virus infection is one of the most serious threats to human health and lives. Some well-known pathogenic viruses include Variola virus, Flu virus, Ebola virus, Zika virus, Severe Acute Respiratory Syndrome Coronavirus (SARS-CoV), and Middle East Respiratory Syndrome Coronavirus (MERS-CoV). Currently, the global pandemic of Coronavirus Disease 2019 (COVID-19), caused by a novel coronavirus SARS-CoV-2, has triggered enormous human casualties and serious economic losses worldwide. Therefore, it is highly urgent to find effective therapeutic drugs to combat against COVID-19 and save people’s lives. Valinomycin has been demonstrated as a potent antiviral agent against a broad spectrum of viruses such as human coronaviruses, bunyaviruses, enteroviruses, and flavivirus [53]. In 1999, an initial test of valinomycin on the vesicular stomatitis virus (VSV) in Vero cells was performed [42]. It was found that adding 10 μM valinomycin to VSV infected cells led to a 90% decrease in viral titer within 12 h after infection. After the outbreak of SARS in 2003, Wu and colleagues extensively screened more than 10,000 compounds to identify effective anti-SARS agents using a cell-based assay with SARS virus and Vero E6 cells [54]. Notably, valinomycin was found to be the most potent inhibitor among all tested compounds with an EC_50_ value of 0.85 μM, which is 4 times lower than that of the second effective compound reserpine (EC_50_ = 3.4 μM). In another antiviral study, from a library of 502 compounds, valinomycin also exhibited the highest inhibition (IC_50_ = 24 nM) on the replication of porcine reproductive and respiratory syndrome virus (PRRSV) in infected monkey embryonic kidney epithelial (MARC)-145 cells [55]. Recently, different groups have demonstrated the inhibitory effect of valinomycin on MERS-CoV [56,57,58]. When Vero B4 cells were infected by MERS-CoV and treated with 5 μM of valinomycin, the replication of MERS-CoV was remarkably reduced by up to 1000-fold at 48 h post-injection. A further dose-effect relationship experiment indicated that valinomycin was highly potent against MERS-CoV with an IC_50_ value of 84 nM [56]. Meanwhile, another study evaluated MERS-CoV infected Vero E6 cells and found that valinomycin was able to inhibit the virus replication with an EC_50_ of 6.07 μM [58]. A recent study further demonstrated the anti-MERS-CoV activity of valinomycin and the IC_50_ value was determined to be as low as 5 nM using infected Vero E6 cells [57]. In the same study, valinomycin was also shown to be effective against another human coronavirus (HCoV-229E, IC_50_ = 67 nM), Zika virus (IC_50_ = 78 nM), and five other viruses (e.g., bunyaviruses and enteroviruses, IC_50_ values ranged from 41 to 971 nM) [57]. Taken together, these findings demonstrated that valinomycin possesses broad-spectrum antiviral activity. Virus genome sequencing and analysis has revealed that the genome sequence of SARS-CoV-2 is closely related to SARS-CoV (79.6% identity) and MERS-CoV (50% identity) [59,60]. Notably, the amino acid sequences of the seven conserved replicase domains in ORF1ab that are used for CoV species classification are 94.4% identical between SARS-CoV-2 and SARS-CoV [60]. In addition, a recent study reported that valinomycin shows high binding energies towards SARS-CoV-2 proteins by molecular docking and dynamic simulations [61]. Therefore, valinomycin has the potential to be developed as a potent antiviral agent to combat the ongoing, fast-spreading global COVID-19 pandemic.

Possible mechanisms of valinomycin against viruses were also described. In the example of anti-VSV infection, the viral envelope glycoprotein (G protein) was not fully processed in valinomycin-treated cells, making G protein oligosaccharides sensitive to endo-β-*N*-acetylglucosaminidase H cleavage. As a result, most of the oligosaccharides in VSV G protein were not converted to their mature forms, leading to the failure of transport of G protein to the cell surface and its further incorporation into budding viral particles [42]. In another way, valinomycin inhibited the activity of the viral ribonucleoprotein (vRNP) that is responsible for directing viral RNA genome replication and gene transcription [62]. In addition, Sandler et al. [57] also reported that valinomycin significantly blocked virus replication and reduced the number of viral genomes by >90%. This is likely due to the disruption of cellular K^+^ gradient, which is a conserved and critical host factor in virus replication [57]. The antiviral effect of valinomycin can be strongly attenuated if the external medium was supplemented with excess potassium ions (KCl) [63].

### 2.4. Insecticidal and Antiparasitic Activity

Insects and parasites are often vectors that cause human diseases such as the two well-known infectious illnesses malaria and dengue [64]. Valinomycin has been shown to have insecticidal, nematocidal, and antiparasitic activity as well [65,66,67,68]. Angus reported that free ingestion of valinomycin by fifth-instar larvae *Bombyx mori* (approximately 5 μg per larva) caused cessation of feed intake followed by sluggishness and finally paralysis of larvae [69]. The toxicity of valinomycin on the insects *Musca domestica* (house fly) and *Periplaneta americana* (cockroach) was tested and the results indicated that valinomycin was quite toxic to both insects [70]. Flies and cockroaches behaved abnormally at 24 h after injection, appearing sluggish and motor paresis. Interestingly, the toxic effect of valinomycin was found to be dependent on sex, as females of both species were more resistant than males. The half-lethal dose (LD_50_) for male flies was 0.02 μg (1.18 μg/g of body weight). Female flies showed a slightly lower susceptibility to valinomycin with a LD_50_ value of 0.03 μg (1.5 μg/g of body weight). In cockroaches, the LD_50_ values for males and females were 0.19 μg (0.25 μg/g of body weight) and 0.5 μg (0.5 μg/g of body weight), respectively [70]. Insecticidal activity bioassays also demonstrated that valinomycin yielded half-lethal concentrations (LC_50_) of 2–3 pm (2–3 μg/mL) for mosquito larvae (*Aedes aegypti*) after 36 h, 3 ppm for two-spotted spider mites (*Tetranychus urticae*), and 35 ppm for Mexican bean beetle larvae (*Epilachna varivestis*) [65]. Gumila et al. screened 22 ionophore compounds for their antimalarial activities and found that valinomycin was active against the growth of *Plasmodium falciparum*, a malaria-causing parasite, with an IC_50_ of 5.3 ng/mL [71]. In addition, valinomycin exhibited significant inhibitory activity against the parasites *Leishmania major* (IC_50_ < 0.11 μM) and *Trypanosoma brucei brucei* (IC_50_ 0.0032 μM), which cause leishmaniasis and African sleeping sickness, respectively [68]. Another study investigated the effect and the mechanism of action of valinomycin on *Babesia gibsoni*, a blood parasite that causes hemolytic anemia in dogs [72]. The authors found that the antibabesial activity of valinomycin depended on the potassium concentration in canine erythrocytes. In erythrocytes with low potassium, the IC_50_ value was calculated as 2.32 ng/mL. However, a high concentration of potassium in canine erythrocytes notably impaired the antibabesial activity of valinomycin with an increasing IC_50_ value of 570 ng/mL. Furthermore, the effect of valinomycin on *B. gibsoni* almost disappeared in culture media containing high concentrations of potassium. The mechanism of action was suggested that valinomycin may destroy *B. gibsoni* by changing the intracellular potassium concentration, which might be maintained by an active transporter. However, the cation (K^+^) transporter of *B. gibsoni* was not identified in the study.

### 2.5. Antitumor Activity

Antitumor activity is another important bioactivity of valinomycin and its antitumor efficacy has been evaluated against multiple tumor cell lines [42,73,74,75,76,77,78]. The half-maximal growth inhibition (GI_50_) concentrations of valinomycin against six human tumor cells (e.g., ovary OVCAR-3, lung NCI-H460, and renal A-498) were determined to be at nanogram levels, ranging from 0.19 to 1.9 ng/mL [42]. The antitumor mechanism of valinomycin is primarily based on the induction of cell apoptosis/death through a couple of different pathways. Valinomycin-treated rat ascites hepatoma cells (AH-130) underwent several typical apoptotic events including the loss of mitochondrial membrane potential, caspase-3 activation, DNA fragmentation, cell shrinkage, and formation of pycnotic nucleus [76]. Under a hypoglycemic growth condition (glucose starvation), valinomycin strongly inhibited the transcription and translation of glucose-regulated protein 78 (GRP78), a molecular chaperone associated with a stress-signaling pathway in tumor cells, which induced selective cell death of glucose-starved HT-29 human colon carcinoma cells [77]. Valinomycin was also found to inhibit human neuronal glioblastoma cells. Similar to non-neuronal cells, glioblastoma cells were sensitive to K^+^-efflux mediated apoptosis, which was induced by valinomycin, resulting in a dramatic depletion of intracellular K^+^, significant depolarization of mitochondria, and obvious cell shrinkage. Meanwhile, valinomycin increased caspase-3 activation and reduced the expression of an anti-apoptotic protein Bcl-2. In addition, valinomycin stimulated a nuclear translocation of the apoptosis-inducing factor (AIF), which represents a caspase-independent apoptotic pathway in glioblastoma cells [73]. Despite its promising antitumor activity, valinomycin can also induce apoptosis of human natural killer (NK) cells [79,80] and of many other mammalian cell types [81,82,83], making it potentially cellular toxic and consequently it is currently not approved for clinical use. However, the toxic effect of valinomycin on normal cells of the host could be significantly reduced by incorporation of valinomycin in liposomes while maintaining or even enhancing its antitumor activity against the P388 mouse leukemia [75]. Moreover, the low toxic liposomal valinomycin displayed synergistic cytotoxicity against human ovarian carcinoma cells (CaOV-3) when used in combination with the antitumor drug cisplatin [74]. Alternatively, modifying chemical structure resulted in the generation of valinomycin derivatives with a low toxicity. For example, the isopropyl side chains of d-α-hydroxyisovalerate, d-valine, and l-valine were hydroxylated, respectively, yielding hydroxyl valinomycin analogs (Figure S1). While hydroxyl analogs showed less antitumor activity than the parent valinomycin to different extents, they were still pharmacologically significant [84]. Taken together, valinomycin can be a promising candidate for combating a range of human tumors if its overall cytotoxicity could be reduced. Therefore, tailoring valinomycin derivatives with well-defined properties by semi-synthesis or biosynthesis and developing specific valinomycin delivery systems may be reasonable routes to achieve this goal.

**Table 1:** Summary of valinomycin bioactivities.

Bioactivity	Efficacy ^a^	Reference
**Antibacterial**		
*Streptococcus pyogenes*	MIC 0.02 μg/mL	[44]
*Clostridium sporogenes*	MIC 8 μg/mL	[44]
*Enterococcus faecalis*	MIC 0.39–0.78 μg/disk	[42]
*Streptococcus pneumoniae*	MIC 0.39–0.78 μg/disk	[42]
*Micrococcus luteus*	MIC 25–50 μg/disk	[42]
**Antifungal**		
*Candida albicans*	MIC 0.39–0.78 μg/disk	[42]
*Cryptococcus neoformans*	MIC 50–100 μg/disk	[42]
*Phytophthora capsici*	IC_50_ 15.9 μg/mL	[47]
*Botrytis cinerea*	MIC 4 μg/mL	[48]
*Magnaporthe grisea*	MIC 4 μg/mL	[48]
*Candida albicans*	MIC 32 μg/mL	[48]
*Colletotrichum gloeosporioides*	MIC 256 μg/mL	[48]
*Rhizoctonia solani*	MIC 256 μg/mL	[48]
*Penicillium verrucosum*	IC_50_ 0.005 ng/mL	[51]
**Antiviral**		
Vesicular stomatitis virus (VSV)	GI_90_ 10 μM	[42]
Severe acute respiratory syndrome coronavirus (SARS-CoV)	EC_50_ 0.85 μM	[54]
Porcine reproductive and respiratory syndrome virus (PRRSV)	IC_50_ 24 nM	[55]
Respiratory syncytial virus (RSV)	IC_50_ 0.0015 μM	[63]
Middle East respiratory syndrome coronavirus (MERS-CoV)	IC_50_ 84 nM	[56]
MERS-CoV	EC_50_ 6.07 μM	[58]
MERS-CoV	IC_50_ 5 nM	[57]
Human coronavirus OC43 (HCoV-OC43)	EC_50_ 4.43 μM	[58]
Human coronavirus NL63 (HCoV-NL63)	EC_50_ 1.89 μM	[58]
Mouse hepatitis virus A59 (MHV-A59)	EC_50_ 6.78 μM	[58]
La Crosse virus (LACV)	IC_50_ 588 nM	[57]
Rift Valley fever virus MP12 (RVFV MP-12)	IC_50_ 41 nM	[57]
Human rhinovirus 2 (HRV2)	IC_50_ 610 nM	[57]
Coxsackievirus B3 (CVB3)	IC_50_ 971 nM	[57]
Zika virus (ZIKV)	IC_50_ 78 nM	[57]
Keystone virus (KEYV)	IC_50_ 156 nM	[57]
Human coronavirus 229E (HCoV-229E)	IC_50_ 67 nM	[57]
Lassa virus (LASV)	EC_50_ 0.61 μM	[62]
Lymphocytic choriomeningitis virus (LCMV)	EC_50_ 0.15 μM	[62]
**Insecticidal**		
*Musca domestica* (male)	LD_50_ 0.02 μg	[70]
*Musca domestica* (female)	LD_50_ 0.03 μg	[70]
*Periplaneta americana* (male)	LD_50_ 0.19 μg	[70]
*Periplaneta americana* (female)	LD_50_ 0.5 μg	[70]
*Aedes aegypti*	LC_50_ 2–3 μg/mL	[65]
*Tetranychus urticae*	LC_50_ 3 ppm	[65]
*Epilachna varivestis*	LC_50_ 35 ppm	[65]
*Plasmodium falciparum*	IC_50_ 5.3 ng/mL	[71]
*Babesia gibsoni* (in low potassium erythrocytes)	IC_50_ 2.32 ng/mL	[72]
*Babesia gibsoni* (in high potassium erythrocytes)	IC_50_ 570 ng/mL	[72]
*Leishmania major*	IC_50_ <0.11 μM	[68]
*Trypanosoma brucei brucei*	IC_50_ 0.0032 μM	[68]
**Antitumor**		
Human ovarian tumor cells CaOV-3	IC_50_ 0.1 nM	[74]
Murine P388 leukemia cancer cells	GI_50_ 0.019 μg/mL	[42]
Human ovary OVCAR-3 tumor cells	GI_50_ 1.9 × 10^−4^ μg/mL	[42]
Brain SF-295 tumor cells	GI_50_ 3.5 × 10^−4^ μg/mL	[42]
Renal A-498 carcinoma cells	GI_50_ 1.9 × 10^−3^ μg/mL	[42]
Lung NCI-H460 cancer cells	GI_50_ 2.1 × 10^−4^ μg/mL	[42]
Colon KM20L2 carcinoma cells	GI_50_ 2.7 × 10^−4^ μg/mL	[42]
Melanoma SK-MEL-5 cancer cells	GI_50_ 2.6 × 10^−4^ μg/mL	[42]
Rat C6 glioma cells	IC_50_ 0.0004 μM	[84]
Human A2780 ovarian carcinoma cells	IC_50_ 2.18 μM	[84]
Human MCF-7 breast carcinoma cells	IC_50_ 1.77 μM	[84]
Human HepG2 liver hepatocellular carcinoma cells	IC_50_ 0.0008 μM	[84]
Human U251 glioma cells	IC_50_ 7.6 nM	[85]

^a^ The values of efficacy are as reported in the references. Note that the molecular weight of valinomycin is 1111.3 g/mol and 1 μg/mL of valinomycin equals to 0.9 μM of valinomycin. MIC, minimum inhibitory concentration; IC_50_, half-maximal inhibitory concentration, EC_50_, half-maximal effective concentration; LD_50_, half-lethal dose; LC_50_, half-lethal concentration; GI_90_, 90% growth inhibition concentration; GI_50_, half-maximal growth inhibition concentration.

## 3. Biogenesis of Valinomycin

After valinomycin was discovered from *S*. *fulvissimus* in 1955, researchers attempted to study the biogenesis of valinomycin. As a first step for this purpose, MacDonald initially investigated possible precursors for valinomycin biosynthesis [86]. To do this, three isotopic compounds (i.e., dl-valine-1-^14^C, d-valine-1-^14^C, and l-valine-1-^14^C) were individually added to the cultivation medium. The results indicated that both d- and l-valyl portions of valinomycin were equally labelled from l-valine-1-^14^C rather than d-valine-1-^14^C in the medium. A lower radioactivity was also found from the d-α-hydroxyisovaleryl portion, however, no activity was detected in the l-lactyl portion of valinomycin. Later, MacDonald and Slater [87] further reported that d-α-hydroxyisovaleric-1-^14^C directly supplied in the medium was incorporated specifically into the corresponding portion of valinomycin but the l-isomer was not incorporated. The authors also pointed out that α-ketoisovaleric acid was not a necessary precursor, however, may be converted to d-α-hydroxyisovaleric acid prior to incorporation. In order to figure out the precursor for the l-lactyl portion of valinomycin, different groups purified responsible enzymes and tested their activities towards possible precursors. Ristow and coworkers [88] found that a partially purified enzyme fraction was able to synthesize valinomycin from l-valine using l-alanine or l-threonine as a precursor of lactic acid. While both ^14^C-labeled amino acids were identified from the l-lactyl moieties of valinomycin, l-threonine was incorporated more efficiently than l-alanine into the l-lactyl portion. The authors proposed that lactic acid could be formed from l-alanine via deamination and reduction, however, the mechanism for transforming l-threonine to lactic acid remained unknown. They also tested if the enzyme complex could directly activate lactic acid, but no activation was observed. By contrast, Anke and Lipmann [89] reported that lactic acid was easily, but l-alanine poorly, incorporated into the l-lactyl moieties of valinomycin. Meanwhile, both groups showed that pyruvate was not able to be activated by their own purified enzyme fractions. Taken together, early studies all agreed that l-valine was a precursor for both l- and d-valyl portions in valinomycin. However, it remained unclear whether the hydroxyl acids like lactic acid were direct precursors or transformed from upstream amino acids (e.g., l-alanine) for the synthesis of valinomycin. The results from early studies thus were ambiguous and partially contradicted with each other.

In 1990, the genetic loci involved in valinomycin biosynthesis were identified within a 120 kb region of chromosomal DNA from *Streptomyces levoris* A-9; however, the exact boundary of each necessary gene was not determined [90]. After more than a decade, the complete gene cluster responsible for valinomycin biosynthesis (termed as *vlm* gene cluster) in *Streptomyces tsusimaensis* ATCC 15141 was cloned, sequenced, and partially characterized by Cheng [91]. Sequence analysis indicated that the *vlm* gene cluster (>23 kb) encompassed seven open reading frames (ORFs), of which *vlm1* (10,287 bp) and *vlm2* (7968 bp) encode two distinct NRPSs Vlm1 and Vlm2, respectively. Vlm1 and Vlm2 together constituted valinomycin synthetase, which is classified as an iterative type B NRPS for valinomycin biosynthesis. According to the protein sequence analysis, Cheng proposed that valinomycin synthetase was a four-module NRPS system with sixteen distinctive domains [91]. Vlm1 and Vlm2 comprised modules 1/2 and modules 3/4, respectively. This modular organization was consistent with the assembly of the tetradepsipeptide basic unit d-α-hydroxyisovaleryl-d-valyl-l-lactyl-l-valyl. Module 1 was predicted to be responsible for d-α-hydroxyisovaleric acid (d-Hiv) incorporation containing two hypothetical domains transaminase (TA) and dehydrogenase (DH_2_). d-Hiv was postulated to be transformed from l-valine (l-Val) by sequential transamination (catalyzed by TA to form α-ketoisovalerate (Kiv)) and dehydrogenation (catalyzed by DH_2_ to yield d-Hiv). In module 2, an epimerase (E) domain was identified for the conversion of l-Val to d-Val. A hypothetical DH_2_ domain was also suggested to embed in module 3 that acts as a l-lactate (l-Lac) dehydrogenase to convert pyruvate (Pyr) to l-Lac. The final module 4 was proposed being responsible for l-Val incorporation. A thioesterase (TE) domain located at the end of module 4 catalyzing a head-to-tail cyclization to form the mature product valinomycin. Soon after, Magarvey et al. [92] independently sequenced the *vlm* gene cluster from *S. levoris* A-9 but no TA domain was found. Instead, reductase domains were found within Vlm1 and Vlm2. As a result, the domain organization of valinomycin synthetase was rationally modified based on the above proposed model by Cheng [91]. Specifically, modules 1 and 3 each contained a ketoreductase (KR) domain, but not TA/DH_2_, being responsible for the reduction of Kiv to d-Hiv and Pyr to l-Lac, respectively. However, this assumption solely depended on sequence homologies to similar NRPS clusters and was not proven by further experiments in the study [92].

**Figure 3:**
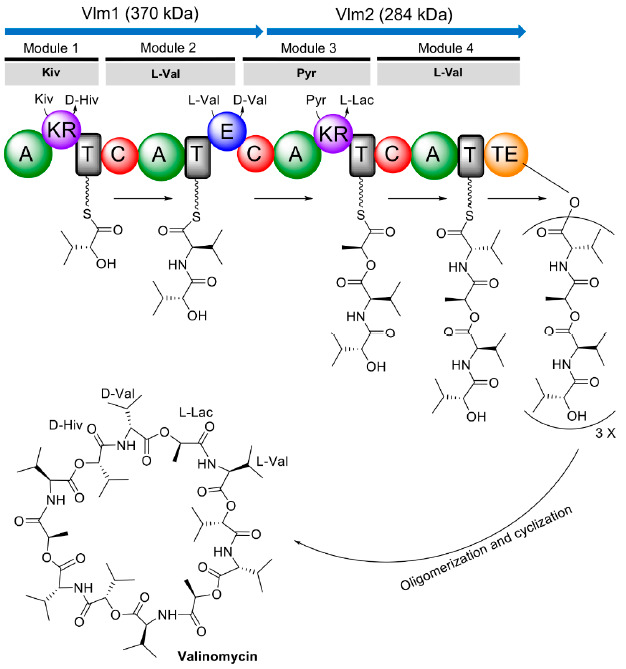
Proposed domain organization of valinomycin synthetase and valinomycin biosynthesis. Kiv, ketoisovalerate; d-Hiv, d-hydroxyisovalerate; l-Val, l-valine; d-Val, d-valine; Pyr, pyruvate; l-Lac, l-lactate; A, adenylation domain; KR, ketoreductase domain; T, thiolation domain; C, condensation domain; E, epimerase domain; TE, thioesterase domain.

Until 2014, Jaitzig and colleagues provided the first experimental proof for the previous proposed model of valinomycin synthetase [93]. The authors purified Vlm1 and Vlm2 from a heterologous expression host *E. coli* and performed an in vitro enzymatic activity assay towards possible substrates. The results indicated that both Vlm1 and Vlm2 activated l-Val, which has been demonstrated as a substrate for valinomycin biosynthesis in the early studies. By contrast, the activation of d-Val was not observed. Importantly, two keto acids Kiv and Pyr were selectively activated by Vlm1 and Vlm2, respectively. However, other tested hydroxyl acids l/d-Hiv and l/d-Lac were not activated by both NRPSs. Therefore, the direct substrates of valinomycin were concluded to be l-Val, Pyr, and Kiv. This also confirmed the correctness of the proposed valinomycin synthetase model by Magarvey et al. [92]. As shown in Figure 3, module 1 activates Kiv, which is subsequently reduced to d-Hiv by a dedicated KR domain; l-Val is activated by module 2 and an E domain in this module converts l-Val to d-Val; another KR domain in module 3 reduces activated Pyr to l-Lac; and the fourth module activates l-Val. Collectively, the four modules of valinomycin synthetase are iteratively reused to assemble three tetradepsipeptide basic units of d-α-hydroxyisovaleryl-d-valyl-l-lactyl-l-valyl, which are finally oligomerized and macrolactonized by a C-terminal TE domain to form the 36-membered cyclododecadepsipeptide valinomycin. Recently, the structure of the TE domain was solved and the mechanism how TE oligomerizes and cyclizes a linear full-length dodecadepsipeptidyl intermediate was elucidated [94]. However, the structure of the whole NRPS valinomycin synthetase (Vlm1 and Vlm2) has not been reported up to now.

## 4. Biosynthesis of Valinomycin in Native Producers

Native producers of valinomycin are mostly *Streptomyces* species that have been isolated from various environments including soil, desert, food, plants, feces, indoor air, and marine sponges [42,47,48,51,65,68,90,91,95,96]. Interestingly, several *Bacillus* strains isolated from *Brassica* seeds and plants were also found to produce valinomycin. Notably, 12 out of 14 *Bacillus pumilus* isolates were able to synthesize valinomycin. In addition, valinomycin was detected from the culture extracts of *Bacillus amyloliquefaciens* (3 out of 18 isolates) and *Bacillus subtilis* (1 out of 19 isolates) as well [97]. More interestingly, the gene cluster for valinomycin biosynthesis was also identified from a *Rothia nasimurium* strain isolated from a porcine tonsil [98]. The occurrence of valinomycin in many *Streptomyces* strains is interesting. Matter et al. [99] studied the conservation, ecology, and evolution of *vlm* gene clusters from eight valinomycin-producing strains originated from North America, Europe, and Asia. They found that the DNA sequences of *vlm* gene cluster were highly conserved among all eight strains. According to the phylogenetic relationships of these strains, the evolution of *vlm* gene cluster represented a vertical transmission (VT) pattern rather than horizontal gene transfer (HGT), which often happens in the transmission of natural product biosynthetic gene clusters [100].

Currently, more than 20 *Streptomyces* strains have been reported to produce valinomycin, albeit their productivities are very different as summarized in Table 2. Production of valinomycin using native producers is still limited because, for example, the cultivation of *Streptomyces* microorganisms is time-consuming (several days) and the genetic manipulation of *Streptomyces* is complex and laborious. Only a few studies optimized different conditions for enhanced production of valinomycin using native *Streptomyces* strains. An orchard soil isolated strain *Streptomyces* sp. M10 was capable of synthesizing valinomycin yet with a relative low yield (about 3.83 mg/L) [48]. A later study discovered that, in addition to valinomycin, this M10 strain also produced bafilomycin (Figure S2), which shares a common precursor (α-ketoisovaleric acid, Kiv) with valinomycin [101]. By disrupting the bafilomycin biosynthetic genes, the yield of valinomycin was increased up to 6 mg/L, which is 1.5-fold higher than the productivity of the parental strain. Further disruption of the branched-chain α-keto acid dehydrogenase (BCDH) gene clusters, encoding a BCDH enzyme complex that can convert Kiv to isobutyric acid, valinomycin production in the mutant was enhanced to 16 mg/L. Another optimization process was performed with a psychrotrophic strain *Streptomyces lavendulae* ACR-DA1, isolated from the soil of a high altitude cold desert [96]. Production of valinomycin was optimized for different fermentation conditions like medium, temperature, and addition of precursors. Finally, the maximum yield of valinomycin (84 mg/L) was obtained with a synthetic mineral base starch (MBS) medium and precursor addition (l-valine) at a low temperature of 10 °C for a period of eight-day cultivation in shake flasks. However, the yield was reduced to 19.4 mg/L when the fermentation was scaled up to a 5 L bioreactor level [102].

## 5. Reconstituted Biosynthesis of Valinomycin In Vivo and In Vitro

### 5.1. Heterologous Production of Valinomycin in Escherichia coli

Although valinomycin is a *Streptomyces* originated natural compound, there is so far no report on reconstituted biosynthesis of valinomycin in *Streptomyces* hosts, which have been well-developed for heterologous expression of natural product gene clusters [103,104]. During the past decades, *E. coli* has emerged as a powerful surrogate host for heterologous production of intricate natural products by recombinant expression of their entire gene clusters in the host cells [105,106,107,108]. The choice of *E. coli* as a heterologous producer is due to its remarkable advantages: simple cultivation conditions, fast growth rate, extensive genetic tools, well-understood native metabolic networks, and easy scale-up fermentation processes. To establish a robust cell factory for heterologous production of valinomycin, the Neubauer group at Technische Universität Berlin intensively utilized *E. coli* to achieve this goal with multiple strategies from strain improvement to bioprocess engineering. In this section, we thus summarize recent reports on heterologous production of valinomycin in *E. coli* (valinomycin yields are listed in Table 2).

Prior to reconstituted biosynthesis of valinomycin in *E. coli*, at least two challenging questions have to be answered. First, is the heterologous host capable of expressing correctly folded and posttranslationally modified NRPSs (i.e., Vlm1 and Vlm2)? Both enzymes Vlm1 and Vlm2 are large multimodular NRPSs with molecular weights of 370 and 284 kDa, respectively. Therefore, heterologous expression of such megaenzymes individually or together would be a heavy metabolic burden on host cells. In addition, a prerequisite for the synthesis of valinomycin is posttranslational phosphopantetheinylation of *apo*-T (thiolation) domains in Vlm1 and Vlm2 by a phosphopantetheinyl transferase (PPTase) [109]. This modification of *apo*-T domains generates functional, *holo*-T domains and consequently leads to active *holo*-NRPS [5]. Second, what are precursors of valinomycin and is the host chassis able to provide the building blocks for valinomycin biosynthesis? Early studies in 1960s and 1970s did not unambiguously confirm all precursors of valinomycin except the one l-valine [86,87,88,89]. Although in 2006 a rational model of valinomycin synthetase was proposed and possible substrates were predicted, no experimental evidence to prove this assumption [91,92].

**Figure 4:**
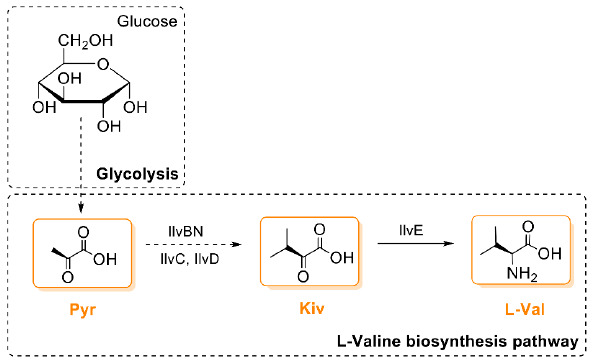
The biosynthetic pathway of valinomycin precursors in *E. coli*. IlvBN, acetohydroxy acid synthase I; IlvC, acetohydroxy acid isomeroreductase; IlvD, dihydroxy acid dehydratase; IlvE, branched chain amino acid aminotransferase; Pyr, pyruvate; Kiv, ketoisovalerate; l-Val, l-valine.

As a first step towards this goal, Jaitzig and colleagues [93] initially cloned two valinomycin NRPS genes *vlm1* (~10 kb) and *vlm2* (~8 kb) from the native producer *S. tsusimaensis* genomic DNA. Soluble expression of Vlm1 or Vlm2 in *E. coli* was successful, enabling the purification of both NRPSs to perform in vitro enzymatic activity and substrate specificity testing. Like other NRPSs, adenylation (A) domains in Vlm1 and Vlm2 are responsible for the selection and activation of dedicated substrates. The substrate activation catalyzed by A domains does not depend on *holo*-T domains and, therefore, the reaction can be assayed with *apo*-NRPSs. With purified Vlm1 and Vlm2, the substrates of valinomycin were experimentally confirmed to be Pyr, Kiv, and l-Val (for details, see the description in the section of “3. Biogenesis of Valinomycin”). However, *apo*-NRPSs have to be functionalized by PPTase to catalyze valinomycin formation. To this end, the *sfp* gene from *Bacillus subtilis* encoding a promiscuous PPTase was integrated into the *E. coli* genome. Finally, coexpression of Vlm1 and Vlm2 in the engineered *E. coli* host, which can directly provide three substrates from its own primary metabolism (i.e., the glycolysis and the branched chain amino acid l-valine biosynthesis pathway; see Figure 4), allowed autonomous formation of valinomycin without substrate feeding. While recombinant production of valinomycin in *E. coli* was successful, only a relatively low yield was obtained (0.3 mg/L) from a batch cultivation using the Terrific Broth (TB) medium. This motivates further optimization to achieve high valinomycin yields.

The initial low yield of valinomycin was ascribed to the batch cultivation format [93]. Once the nutrients in TB medium were depleted, the growth of *E. coli* gradually ceased that limited the supply of valinomycin substrates. To overcome this limitation, *E. coli* cultivations were subsequently switched from a batch to an enzyme-controlled fed-batch-like glucose delivery (EnBase^®^) system in shake flasks [110]. This fed-batch-like cultivation system enabled a prolonged cell growth phase and high cell densities by a controlled continuous internal supply of glucose, which is gradually released by a biocatalyst from the dissolved glucose polymer [111,112]. With this fed-batch cultivation mode, valinomycin yields were significantly increased from 0.3 to 2.4 mg/L. A subsequent design of experiment (DoE)-driven optimization further improved the yield up to 6.4 mg/L. Notably, repeated glucose polymer feeding to the cultivation system not only resulted in flask-scale high cell densities (a final OD_600_ of 55) but also enhanced valinomycin production to a yield of 10 mg/L, which is a 33-fold increase as compared to the initial yield obtained with TB batch cultivations [110].

**Figure 5:**
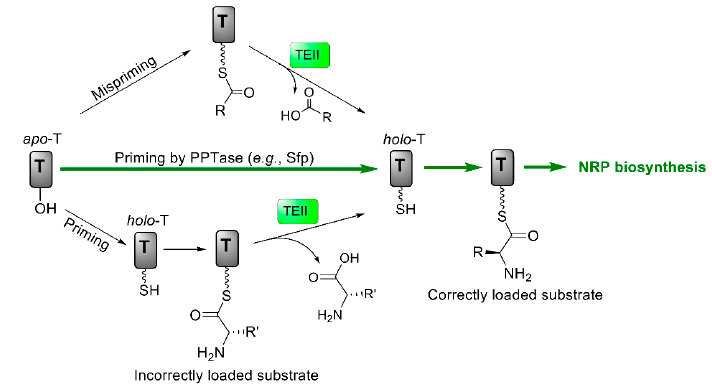
Regeneration of the functionality of T domains catalyzed by type II thioesterase (TEII). T, thiolation domain; PPTase, phosphopantetheinyl transferase; NRP, nonribosomal peptide.

While two core NRPS genes in the valinomycin biosynthetic gene cluster are *vlm1* and *vlm2*, other five genes are also identified in the gene cluster and one of them encodes a discrete type II thioesterase (TEII) [91]. In general, TEII in NRPS gene clusters serves as a repair (editing) enzyme to restore the functionality (activity) of NRPS through hydrolysis of either misacylated thiol groups or incorrectly loaded substrates on the T domains (Figure 5) [113,114,115]. However, whether this editing enzyme TEII could keep the activity of valinomycin synthetase in a heterologous host remained unknown. To test its dedicated function, the cognate TEII was coexpressed with Vlm1 and Vlm2 in *E. coli* for valinomycin production [116]. By doing this, valinomycin yields were significantly improved from 0.5 (without TEII coexpression) to 3.3 mg/L, demonstrating the reconstitutive function of TEII in heterologous valinomycin biosynthesis. A following enzyme-based fed-batch cultivation system in shake flasks finally gave rise to the highest production of valinomycin (13 mg/L).

Since fed-batch high cell density cultivations benefit valinomycin production in *E. coli*, this cultivation mode was scaled up from 1 mL in 24-well plates to a benchtop bioreactor [117]. An initial cultivation was performed with 2 L culture volume. Although the cell density at the end of the fermentation reached 120 (OD_600_), the valinomycin yield was modest (2 mg/L). Protein expression analysis indicated that Vlm2 was not well expressed in this bioreactor fermentation, which is likely due to plasmid instability. Additionally, a 10 L laboratory scale-down two-compartment reactor (TCR), which can mimic similar situations in large industrial bioreactors, was used to produce valinomycin. The production was not affected by oscillating conditions (i.e., high glucose and oxygen limitation in a feeding zone) in the TCR bioreactor, suggesting that this scale-up strategy based on consistent fed-batch cultivations for bioprocess development may be robust and feasible even for larger production scales. However, there is still space for further optimization to achieve mass production of valinomycin in large-scale fermentations to meet the industrial demand. Such possibilities might arise from different levels, for example, constructing a more stable coexpression system (Vlm1, Vlm2, and TEII), engineering the metabolic pathways of host cells to provide abundant substrates (Pyr, Kiv, and l-Val), and optimizing key large-scale fermentation parameters (e.g., glucose feeding, cell growth rate, and dissolved oxygen, etc.).

### 5.2. In Vitro Total Biosynthesis of Valinomycin

In recent years, in vitro cell-free systems are becoming a promising complementary platform to in vivo cell-based systems for biomanufacturing of various products [118,119,120,121,122]. In this context, cell-free protein synthesis (CFPS), cell-free metabolic engineering (CFME), and coupled CFPS-ME have been used to synthesize value-added chemicals, cellular toxic compounds, and complex natural products, among others [123,124,125,126,127,128,129]. The first example of using CFPS for NRP biosynthesis was a diketopiperazine (DKP) d-Phe-l-Pro, which is a shunt product of gramicidin S, by coexpression of two NRPS enzymes GrsA and GrsB1 in vitro [124]. This work represented a proof-of-concept for how one could apply CFPS to synthesize complex natural products like NRPs.

Recently, total in vitro biosynthesis of valinomycin was achieved by using *E. coli* cell-free systems [130]. The first challenge was active expression of the two large NRPSs Vlm1 (370 kDa) and Vlm2 (284 kDa), although the *E. coli*-based CFPS system is robust to express many types of proteins with molecular weights often less than one hundred kDa [120,121,131]. Initially, individual plasmids harboring the genes *vlm1* and *vlm2* were used as DNA templates to express the two enzymes in CFPS reactions. With cell extracts prepared from *E. coli* BL21 Star (DE3), both enzymes Vlm1 and Vlm2 were separately expressed in full-length as high soluble fractions. Importantly, they could be coexpressed solubly in a single-pot cell-free reaction. Then, the PPTase protein Sfp was coexpressed with Vlm1 and Vlm2, and this cell-free expressed Sfp was able to activate both enzymes for valinomycin biosynthesis. Finally, the target product valinomycin was successfully detected by LC-MS analysis, however, with a low yield of 9.76 μg/L. Next, the repair enzyme TEII, which was earlier found to be able to regenerate the activity of Vlm1 and Vlm2 and enhance valinomycin production in *E. coli* cells [116], was coexpressed with the aforementioned three proteins (Vlm1, Vlm2, and Sfp). Notably, all four proteins were actively expressed in a one-pot CFPS reaction and TEII indeed also improved valinomycin biosynthesis in the in vitro reaction environment. The final yield from this reaction reached 37 μg/L. Taken together, this work demonstrated the robustness of the *E. coli*-based CFPS system that can express the entire valinomycin biosynthetic gene cluster (>19 kb), which contains two large NRPSs (Vlm1 and Vlm2) and one associated editing enzyme (TEII), as well as a heterologous modification enzyme (Sfp). While this was remarkable, valinomycin yields in CFPS reactions were about three orders of magnitude lower than previous in vivo production in *E. coli* [116].

One of the reasons for low valinomycin yields was possibly resource limitations in CFPS caused by coexperssion of the four enzymes. To bypass this constraint, Vlm1 and Vlm2 were first produced in vivo, followed by preparation of two cell lysates each enriched with Vlm1 or Vlm2. Afterwards, valinomycin was synthesized through an approach called cell-free metabolic engineering (CFME) by simple mixing of two cell lysates. However also here, an unexpected low yield of valinomycin was obtained (5.59 μg/L), even if Vlm1 and Vlm2 were enriched in the cell-free biosynthesis reaction. Therefore, other efforts were carried out to optimize the CFME system for enhanced valinomycin production. By investigating the effect of supplemental cofactors (CoA, NAD, and ATP) and the mass ratio of two lysates on valinomycin synthesis, the yield was increased up to 76.9 μg/L at a mass ratio of 3:1 (cell lysate-Vlm1:cell lysate-Vlm2) without cofactor supplementation. Finally, a coupled CFPS-ME system was used to perform a two-phase biosynthesis. In this approach, TEII was expressed by CFPS in the first reaction phase that could be used to restore the activity of Vlm1 and Vlm2 during the second CFME phase. In addition, when enough TEII enzyme was expressed (3 h), glucose (200 mM) was added to the reaction to fuel the CFME process for another 12 h. This strategy dramatically improved valinomycin biosynthesis with a yield of nearly 30 mg/L, which is more than 5000 times higher than the initial CFME yield (5.59 μg/L). This result suggested that (i) CFPS expressed TEII is active to regenerate the activity of in vivo heterologously expressed Vlm1 and Vlm2 and (ii) native enzymes in cell lysates are active to convert glucose to valinomycin substrates (Pyr, Kiv, and l-Val) through the glycolysis and the branched chain amino acid l-valine biosynthesis pathway (Figure 4).

In sum, this work shows an example to use the *E. coli*-based cell-free system for rapid synthesis of valinomycin and production improvement. It should also be noted that valinomycin originates from *Streptomyces* species with a high GC-content *vlm* gene cluster. Therefore, one might wonder if recently developed *Streptomyces*-based CFPS systems [132,133,134] are more suitable to express *vlm* gene cluster than the *E. coli* CFPS system. Especially, with the improvement of expressed protein yields [135], valinomycin biosynthesis in *Streptomyces* CFPS systems is possible and deserves future studies. Given the robustness and flexibility of cell-free systems, expression of the engineered *vlm* gene cluster using CFPS might be easy and fast for the synthesis of valinomycin analogs with potential novel bioactivities, but low cellular toxicity.

## 6. Conclusions and Outlook

Since the discovery of valinomycin in 1955, it has always received broad attentions from biologists, chemists, engineers, and pharmacologists because of its outstanding biological features and functions. Notably, valinomycin, which was initially identified as an antibiotic, also shows a diverse spectrum of bioactivities including antifungal, antiviral, insecticidal, and antitumor activity. In particular, valinomycin exhibits remarkable inhibitory effects on the viruses SARS-CoV and MERS-CoV, which share high genome similarity with SARS-CoV-2 that causes the current global pandemic of COVID-19. Moreover, molecular docking and dynamic simulations have shown that valinomycin shows high binding energies to SARS-CoV-2 proteins [61]. Therefore, valinomycin is a potential compound to be developed as a potent anti-SARS-CoV-2 agent to combat the ongoing, fast-spreading COVID-19 pandemic. In this context, at least mass production of valinomycin is highly desirable for further structure modification to reduce its overall cellular toxicity.

Although many *Streptomyces* strains have been reported to produce valinomycin, these native hosts are not ideal producers due to, for example, time-consuming cultivations (around 10 days) and laborious genetic manipulations. Alternatively, a heterologous host *E. coli* has been engineered for valinomycin production, followed by a bioprocess development to enhance valinomycin yields (from 0.3 to 13 mg/L) [110,116]. In addition, total in vitro biosynthesis of valinomycin has also been established based on an *E. coli* CFPS system. This approach allows for easy control and optimization without the use of living cells, giving rise to a valinomycin yield as high as 30 mg/L [130]. Of note, the valinomycin synthesis rate (mg/L/h) in in vitro systems was the highest as compared to in vivo production systems using both native producers and the heterologous *E. coli* host (Table 2). Previous work has shown that CFPS reaction volumes could be scaled up linearly over a range of six orders of magnitude to 100 L [136]. Moreover, the costs of cell-free systems can be reduced by numerous opportunities like removing expensive reagents (e.g., exogenous tRNAs) and decreasing substrate concentrations (e.g., amino acids) [121]. These technological progresses will make industrialization of cell-free biotechnology with a great potential to produce target products such as, for example, valinomycin.

Looking forward, more researches and developments of valinomycin will continue to expand in the years to come due to its specific properties and bioactivities. Such directions might include, but are not limited to, the generation of valinomycin analogs/derivatives with low cellular toxicity, development of efficient valinomycin delivery system for precisely targeted treatments, and high-yielding production of valinomycin using an easy, inexpensive, and sustainable way.

## Figures and Tables

**Table 2:** Production levels of valinomycin with native and heterologous hosts.

Production	Yield a	Note	Reference
**Native host**			
*Streptomyces fulvissimus*	17 mg/L	Static cultivation in flask, 20 d	[12]
*Streptomyces* sp. PRL1642	50–58 mg/L	Shake-flask, 3–5 d	[86]
*Streptomyces griseus* var. *flexipertum*	N.R.	Bioreactor, 5 d	[65]
*Streptomyces levoris* A-9	N.R.	Shake-flask, 5 d	[90]
*Streptomyces griseus* strains 2/ppi, 8/ppi, 10/ppi, and 1/k	0.6–1.4 mg/mg wet biomass	Cultivation on agar plates, 10–12 d	[95]
*Streptomyces anulatus* (Montana)	4.65 mg/L	Shake-flask, 3 d	[42]
*Streptomyces anulatus* (Malaysian)	1.5 mg/L	Shake-flask, 3 d	[42]
*Streptomyces exfoliatus* (Malaysian)	3.9 mg/L	Shake-flask, 3 d	[42]
*Streptomyces padanus* TH-04	70 mg/L	Shake-flask, 7 d	[47]
*Streptomyces* sp. M10	3. 83 mg/L	Shake-flask, 2 d	[48]
*Streptomyces* sp. M10	16 mg/L	Shake-flask, 3 d, engineered M10 strain	[101]
*Streptomyces tsusimaensis*	8.45 mg/L	Shake-flask, 6 d	[99]
*Streptomyces griseus* 1/k	10.19 mg/L	Shake-flask, 6 d	[99]
*Streptomyces griseus* 10/ppi	22.08 mg/L	Shake-flask, 6 d	[99]
*Streptomyces* sp. 22	N.R.	Cultivation on agar plates, 7 d	[68]
*Streptomyces* sp. 34	N.R.	Cultivation on agar plates, 7 d	[68]
*Streptomyces* sp. P11–23B	0.74 mg/L	Shake-flask, 14 d	[85]
*Streptomyces lavendulae* ACR-DA1	84 mg/L	Shake-flask, 8 d, l-valine feeding	[96]
*Streptomyces lavendulae* ACR-DA1	19.4 mg/L	Bioreactor, 8 d	[102]
*Streptomyces* sp. S8	N.R.	Shake-flask, 14 d	[49]
*Streptomyces parvus*	0.15 mg/g dry biomass	Shake-flask, 5 d	[51]
*Bacillus subtilis*	N.R.	Shake-flask, 3 d	[97]
*Bacillus pumilus*	N.R.	Shake-flask, 3 d	[97]
*Bacillus amyloliquefaciens*	N.R.	Shake-flask, 3 d	[97]
*Rothia nasimurium*	N.R.	Genome sequencing and prediction	[98]
**Heterologous host**			
*Escherichia coli*	0.3 mg/L	Shake-flask, 36 h	[93]
*Escherichia coli*	6.4 mg/L	24-well plate, 48 h	[110]
*Escherichia coli*	10 mg/L	Shake-flask, fed-batch cultivation, 48 h	[110]
*Escherichia coli*	2 mg/L	Bioreactor, 48 h	[117]
*Escherichia coli*	13 mg/L	Shake-flask, 48 h, coexpression of TEII	[116]
**In vitro system**			
*Escherichia coli* cell-free system	37 μg/L	CFPS, 20 h	[130]
*Escherichia coli* cell-free system	77 μg/L	CFME, 12 h	[130]
*Escherichia coli* cell-free system	30 mg/L	CFPS-ME, 15 h	[130]

^a^ Yields are as reported or calculated from reported data. N.R., not reported.

## Supplementary Materials

The following are available online at https://www.mdpi.com/article/10.3390/microorganisms9040780/s1. Figure S1: Valinomycin analogs with a hydroxyl group (OH) at the isopropyl side chain of d-Hiv (left), d-Val (middle), and l-Val (right), respectively. Figure S2: Chemical structure of bafilomycin.
