# Supplementary material for: The Nonribosomal Peptide Valinomycin: From Discovery to Bioactivity and Biosynthesis
Source: Microorganisms. 2021 Apr 8;9(4):780. doi: 10.3390/microorganisms9040780 (PMC8068249; doi:10.3390/microorganisms9040780)
Supplement: Supplementary file 1 [file microorganisms-09-00780-s001.pdf]

Supplementary Information

The Nonribosomal Peptide Valinomycin: From Discovery to Bioactivity and Biosynthesis

Shuhui Huang [1,†], Yushi Liu [1,†], Wan-Qiu Liu [1], Peter Neubauer [2,*] and Jian Li [1,*]

[1] School of Physical Science and Technology, ShanghaiTech University, Shanghai, 201210, China

[2] Chair of Bioprocess Engineering, Department of Biotechnology, Technische Universität Berlin, 13355 Berlin, Germany

[†] These authors contributed equally.

* Correspondence: lijian@shanghaitech.edu.cn (J.L); peter.neubauer@tu-berlin.de (P.N.)

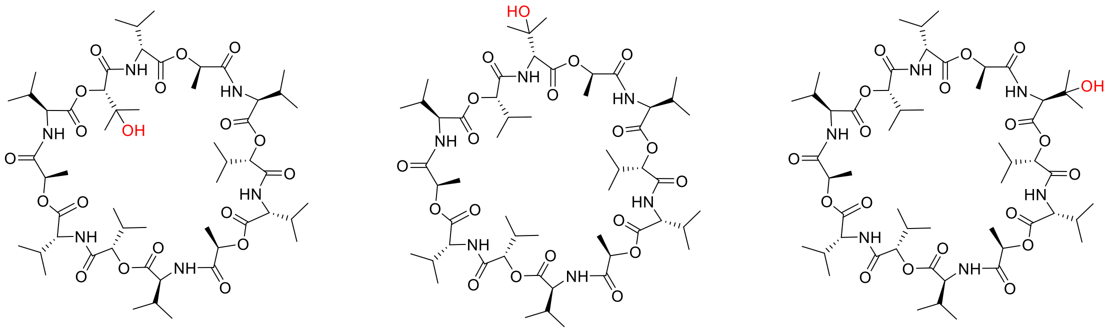

Figure S1. Valinomycin analogs with a hydroxyl group (OH) at the isopropyl side chain of D-Hiv (left), D-Val (middle), and L-Val (right), respectively.

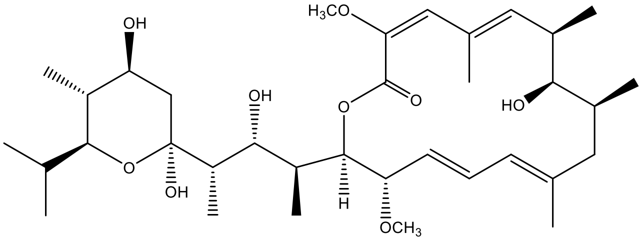

Figure S2. Chemical structure of bafilomycin.
